# Sorafenib Versus Lenvatinib-Based Sequential Systemic Therapy for Advanced Hepatocellular Carcinoma: A Real-World Analysis

**DOI:** 10.3390/cancers14081975

**Published:** 2022-04-13

**Authors:** Catherine Leyh, Ursula Ehmer, Daniel Roessler, Alexander B. Philipp, Florian P. Reiter, Petia Jeliazkova, Leonie S. Jochheim, Matthias Jeschke, Janina Hammig, Johannes M. Ludwig, Jens M. Theysohn, Andreas Geier, Christian M. Lange

**Affiliations:** 1 Department for Gastroenterology and Hepatology, University Duisburg-Essen, 45141 Essen, Germany; catherine.leyh@uk-essen.de (C.L.); leonie.jochheim@uk-essen.de (L.S.J.); matthias.jeschke@uk-essen.de (M.J.); janina.hammig@uk-essen.de (J.H.); 2 Department of Internal Medicine II, Klinikum Rechts der Isar, Technische Universität München, 81675 Munich, Germany; ursula.ehmer@mri.tum.de (U.E.); petia.jeliazkova@mri.tum.de (P.J.); 3 Department of Internal Medicine II, LMU University Hospital Munich, 81377 Munich, Germany; daniel.roessler@med.uni-muenchen.de (D.R.); alexander.philipp@med.uni-muenchen.de (A.B.P.); 4 Department of Medicine II, Division of Hepatology, University Hospital Würzburg, 97080 Würzburg, Germany; reiter_f@ukw.de (F.P.R.); geier_a2@ukw.de (A.G.); 5 Institute of Diagnostic and Interventional Radiology and Neuroradiology, University Duisburg-Essen, 45141 Essen, Germany; johannes.ludwig@uk-essen.de (J.M.L.); jens.theysohn@uk-essen.de (J.M.T.)

**Keywords:** hepatocellular carcinoma, systemic therapy, tyrosine-kinase inhibitor

## Abstract

**Simple Summary:**

An increasing number of new systemic therapies have significantly enhanced treatment opportunities for patients with advanced liver cancer. Yet, it is unclear which sequence of systemic therapies is best for individual patients. In the present study, we compared systemic treatment lines in patients who received either first-line therapy with sorafenib or lenvatinib, two important tyrosine-kinase inhibitors for the treatment of liver cancer. Overall, baseline liver function rather than choice of first-line tyrosine kinase inhibitor (TKI) had an impact on median overall survival (mOS).

**Abstract:**

The optimal treatment sequence of tyrosine kinase inhibitor (TKI)-based therapy in patients with hepatocellular carcinoma (HCC) remains unclear. Therefore, sequential systemic therapy after first-line therapy with sorafenib or lenvatinib was compared in a retrospective real-world cohort. In total, 164 patients with HCC were included. Child B cirrhosis was present in 26 patients (16.5%), whereas 132 patients (83.5%) had preserved liver function. In total, 72 patients (44%) discontinued systemic therapy after first-line therapy while 51 (31%) and 31 (19%) patients received 2 or more treatment lines. Most notably, median overall survival (mOS) was influenced by liver functional status and patient performance status at the beginning of first-line therapy. Patients receiving a sequential therapy regimen had significantly longer mOS compared to patients that discontinued systemic therapy after omitting first-line treatment. The choice of the initial TKI did not impact mOS. A clear deterioration of liver function could be observed during the course of TKI-based treatment.

## 1. Introduction

Hepatocellular carcinoma (HCC) is one of the leading causes of cancer-related mortality worldwide [1]. Systemic therapy is the recommended treatment for patients with advanced-stage HCC (Barcelona Clinic Liver Cancer (BCLC) stage C) and patients with intermediate-stage HCC (BCLC B) not suitable for locoregional treatment [2,3,4]. From 2007 to 2018, the only available systemic therapy was the tyrosine kinase inhibitor (TKI) sorafenib [5]. As a result of several positive phase III studies, multiple new substances have been approved for first-, second-, and third-line therapy in the last few years [6,7,8,9,10].

In 2018, lenvatinib, a multikinase inhibitor (MKI), was approved for first-line therapy as an alternative to sorafenib. Compared to first-line treatment with sorafenib, therapy with lenvatinib resulted in a significant improvement in progression-free survival (PFS), time to progression (TTP), and the objective response rate (ORR). In terms of median overall survival (mOS), lenvatinib was non-inferior to sorafenib [6]. Until 2017, there was no approved option for a second-line therapy in case of failure of the applied first-line therapy. The breakthrough came with the approval of regorafenib, also an MKI. In a phase III trial, which included patients with tumor progression under therapy with sorafenib, a significant increase in mOS was observed for sequential therapy with regorafenib compared to placebo. Good tolerance of sorafenib (≥400 mg/day for ≥20 of the last 28 days of therapy) was an inclusion criterion in the approval study [7]. For patients with tumor progression during treatment with sorafenib and poor tolerability to sorafenib, the MKI cabozantinib was approved as second-line or third-line therapy in 2018. In addition to inhibiting the vascular endothelial growth factor (VEGF) signaling pathway, it also inhibits the tyrosine kinases MET and AXL, which are involved in tumor resistance to sorafenib [8]. In 2019, ramucirumab, a monoclonal antibody targeting the VEGF receptor 2, was approved for patients with tumor progression under sorafenib and elevated alpha-fetoprotein (AFP ≥ 400 ng/mL) [9].

Immune checkpoint inhibitors (ICIs) have recently been added to the armamentarium to treat advanced HCC, especially in combination therapies. For example, the combination of the programmed cell death-ligand 1 (PD-L1) inhibitor atezolizumab and the VEGF antibody bevacizumab significantly improved PFS and OS compared to sorafenib in first-line therapy [10]. This combination therapy has therefore become the new standard of care in first-line therapy for advanced HCC [3]. However, in contrast to the Food and Drug Administration (FDA), the European Medicines Agency (EMA) did not approve pembrolizumab and nivolumab as second-line monotherapy due to the lack of significant benefits in the respective phase III trials [11,12].

Collectively, these exciting developments have significantly improved treatment options and the prognosis of patients with advanced HCC. Yet, optimal algorithms for systemic therapies have not been established, and it remains unclear which is the best sequence for each patient. We therefore performed a retrospective analysis of real-world patients with HCC who received systemic therapy after the approval of lenvatinib.

## 2. Materials and Methods

### 2.1. Study Design and Patients

All consecutive patients with HCC with or without liver cirrhosis, who started first-line systemic therapy with TKIs across four German centers between January 2017 and October 2020 (the approval date of atezolizumab/bevacizumab), were included in this retrospective study [13,14].

Diagnosis of HCC was established in a guideline-directed manner following the recommendations of the European Association for the Study of the Liver (EASL). In patients suffering from liver cirrhosis, diagnosis of HCC was either based on histology or on two distinct contrast-enhanced imaging modalities, e.g., dynamic computed tomography (CT) and contrast-enhanced magnetic resonance imaging (MRI), visualizing typical vascularization patterns. In patients without liver cirrhosis, histological diagnosis was established. Patients were excluded if they were younger than 18 years old or had fibrolamellar HCC or mixed hepatocellular cholangiocarcinoma.

Clinical data were extracted retrospectively from the digital patient file. Demographic data, consisting of age, gender, Eastern Cooperative Oncology Group-Performance Status (ECOG-PS), and etiology of liver cirrhosis, liver function, and tumor characteristics, were recorded. Diagnosis of nonalcoholic steatohepatitis (NASH) was established by a combination of typical findings in ultrasound, assessment of the controlled attenuation parameter value (CAP) and histological findings, when available, or in the presence of metabolic syndrome. Proof of any history of significant alcohol intake and/or viral hepatitis was prohibitive of a diagnosis of NASH. Alcoholic steatohepatitis (ASH) was associated with significant alcohol intake, which was defined as consumption more than 20 g/day for women and more than 30 g/day for men. Hepatitis B virus (HBV) infection was defined by either seropositivity for surface antigen (HBsAg) and/or detection of HBV-DNA. Hepatitis C virus (HCV) infection was made according to the detection of HCV antibodies (Abbott, Des Plaines, IL (if USA), USA) and/or HCV-RNA.

Liver function was measured by the Child-Pugh score (CPS), the albumin-bilirubin (ALBI) score and grade, and the model for end-stage liver disease (MELD) score and was determined at baseline and every change in therapy. Previous local treatment (e.g., resection, transarterial chemo- or radioembolization) was also registered. Treatment-related adverse events were classified according to the Common Terminology Criteria for Adverse Events (CTCAE). An MRI or CT scan to determine tumor response was performed every three months. Tumor response was assessed according to the modified Response Evaluation Criteria in Solid Tumors (mRECIST) criteria.

The study was approved by the local ethics committee of the University Hospital Essen (reference number 21-10097-BO) and performed in accordance with the 1964 Helsinki Declaration.

### 2.2. Statistical Analysis

SPSS Statistics 27 (IBM SPSS Statistics version 27.0, Armonk, NY, USA) and Prism 9 (GraphPad Prism version 9.0, San Diego, CA, USA) were used for the statistical analysis. Continuous variables are presented as a median with an interquartile range (IQR); categorical variables are presented as percentages. The chi-square test or Fisher’s exact test were used to compare categorical variables. To compare continuous variables, the Student’s *t*-test, Mann-Whitney-U-test, Wilcoxon test, and Kruskal-Wallis test were used accordingly. For the correlation analysis, we used the Pearson correlation coefficient. Median overall survival (mOS) after the start of first-line systemic therapy was calculated using the Kaplan-Meier method. Patients who were lost to follow-up or were still on therapy were censored. The log-rank test was used to test for significant differences concerning mOS. The duration of each therapy line was defined as the time period between the start of therapy and discontinuation due to progress or intolerance. To further assess the prognostic value of variables, we performed univariate and multivariate analysis by applying the Cox regression model. The hazard ratio (HR) and 95% confidence interval are given. *p* values are 2-sided and considered significant if <0.05.

## 3. Results

### 3.1. Baseline Characteristics

A total of 164 patients were enrolled in the study. Detailed baseline characteristics of these patients are shown in Table 1. Most patients were male (85%), with a median age of 68 years (IQR 60.25–73). Their general condition, represented by the ECOG-PS, was good or only slightly impaired at baseline (ECOG PS 0 *n* = 92, 57%; ECOG PS 1 *n* = 64, 40%; ECOG PS 2 *n* = 4, 2%; ECOG PS 3 *n* = 1, 1%). Alcoholic steatohepatitis (ASH, *n* = 45, 29%), non-alcoholic steatohepatitis (NASH, *n* = 32, 20%), and viral hepatitis (*n* = 46, 29%) were the most common etiologies of underlying liver disease. Liver function was preserved in the majority of patients (Child-Pugh A, *n* = 132, 83.5%), but 26 patients (16.5%) had Child B cirrhosis. Ascites was diagnosed in 40 patients (25%), including 7 patients (4%) with refractory ascites. In total, 65 patients (42%) and 85 patients (55%) had ALBI grade 1 and 2, respectively. The median ALBI score was −2.47 (IQR −2.91 to −2.06). The median albumin and bilirubin values were 3.9 (IQR 3.5–4.2) and 1.0 (IQR 0.6–1.6), respectively. In total, 80 patients (49%) had extrahepatic tumor manifestations.

BCLC C was the most common tumor stage (BCLC A N = 1, 1%; BCLC B *n* = 57, 35%, BCLC C *n* = 104, 63%, BCLC D *n* = 1, 1%). In total, 49 patients (30%) had a macrovascular invasion. Most patients (*n* = 106, 65%) had received prior locoregional therapy. Transarterial radioembolization (TARE, *n* = 55, 34%) and transarterial chemoembolization (TACE, *n* = 55, 34%) were the most frequently applied locoregional therapies.

### 3.2. Sequences and Durations of Systemic Therapies

**Figure 1:**
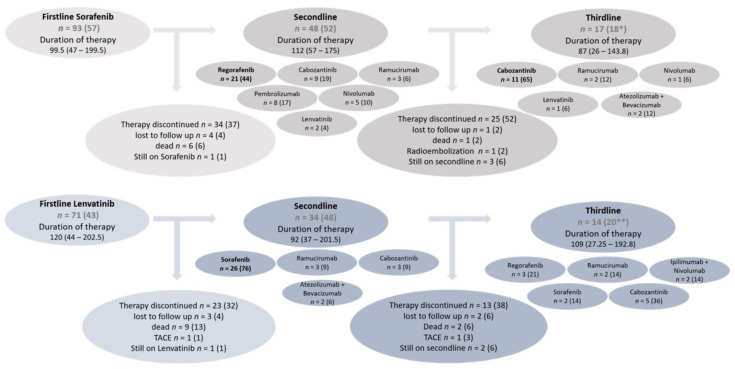
Flowchart of sequential systemic therapies starting either with sorafenib or lenvatinib as first line (*n* (%)); duration of therapy (days, median (IQR)); * refers to *n* = 93 (initial sorafenib cohort); ** refers to *n* = 71 (initial lenvatinib cohort).

Of the 164 patients included in the study, 72 (44%) discontinued systemic therapy after first-line therapy. In total, 51 patients (31%) were treated with a sequential treatment consisting of 2 therapy lines while 31 patients (19%) were treated with more than 2 available agents, with a maximum of 4 sequential treatment lines (*n* = 9, 5%). A summary of the systemic therapy sequences is provided in Figure 1.

The most common first-line systemic therapy was sorafenib (*n* = 93, 57%), followed by lenvatinib (*n* = 71, 43%). The baseline characteristics of patients treated with sorafenib vs. lenvatinib as first-line therapy are shown in Table 1. Patients treated with lenvatinib as first-line presented a slightly better general condition at baseline than those who started systemic treatment with sorafenib (ECOG PS 0 lenvatinib 69% vs. sorafenib 48%, ECOG PS 1 lenvatinib 29% vs. sorafenib 48%, *p* = 0.04). Regarding the etiology, patients in the sorafenib subgroup presented more often with ASH as underlying liver disease compared to those in the lenvatinib sub-group (sorafenib 40% vs. lenvatinib 13%) while patients treated with lenvatinib more often had a diagnosis of chronic hepatitis B compared to patients treated with sorafenib (sorafenib 11% vs. lenvatinib 21%). NASH was equally represented in both cohorts. Other parameters, in particular those reflecting liver function, showed no significant differences between both subgroups

### 3.3. Sequential Treatment after First-Line Therapy with Sorafenib

Of all 93 patients treated with sorafenib as first-line therapy, 40 patients (43%) discontinued systemic therapy during or after first-line treatment (Figure 1). Intolerance of therapy (*n* = 18, 45%), most notably liver function deterioration (*n* = 13/18, 72%), was the most common reason for treatment discontinuation, as shown in Table 2. In total, 6 patients (15%) died during first-line therapy. Patients with tumor progression on first-line treatment with sorafenib usually presented a preserved liver function and received a second-line therapy (*n* = 48, 52%), as shown in Table 2. Regorafenib (*n* = 21, 44%) was the most frequently used TKI as second-line systemic treatment following sorafenib. In total, 13 patients (27%) were treated with an ICI as second-line therapy. In total, 13 patients (18%) received a third-line therapy. In these cases, the most commonly used drug was cabozantinib (*n* = 11, 65%). The median treatment duration of the entire therapy sequence after initiating first-line therapy with sorafenib was 175.5 (IQR 80.5–333) days (Table 3).

### 3.4. Sequential Treatment after First-Line Therapy with Lenvatinib

Of the 71 patients who received first-line systemic therapy with lenvatinib, 32 patients (45%) discontinued systemic therapy during or after first-line therapy (Figure 1). In 16 patients (50%), systemic therapy was discontinued due to intolerance or toxicity (Table 2). Of these, 8 patients (50%) showed a deterioration of liver function. After tumor progression on lenvatinib, 34 patients (48%) received a second-line therapy. Sorafenib was the most commonly used TKI in second-line therapy (n = 26, 76%). In total, 14 patients (20%) were treated with a third-line therapy.

The median treatment duration of the entire sequential therapy after initiating first-line therapy with lenvatinib was comparable to the above described sorafenib subgroup (155 days vs. 175.5 days, respectively, *p* = 0.8), as shown in Table 3.

### 3.5. Deterioration of Liver Function during Systemic Therapy

Liver function at baseline was similar in patients who received first-line therapy with sorafenib versus lenvatinib. Most patients were Child-Pugh score A at baseline (72 patients (83%) with CPS A in the sorafenib group and 60 patients (85%) with CPS A in the lenvatinib group). The median baseline ALBI score was −2.40 (IQR −2.87 to −1.93) and −2.54 (IQR −3.00 to −2.14) in the sorafenib group and lenvatinib group, respectively. The distribution of ALBI grades was similar in both subgroups. At the end of first-line therapy, a significant deterioration of liver function, as represented by the CPS and the ALBI score, could be observed regardless of whether the patients received sorafenib or lenvatinib as first-line therapy (Table 4, Supplementary Materials Figures S4 and S5). CPS deteriorated by about 1 point in both sub-groups from the start to the end of first-line therapy (1.1 and 0.9 points after sorafenib and lenvatinib treatment, respectively). The ALBI score was significantly deteriorated at the end of first-line therapy in both subgroups (−1.67, IQR −2.48 to −1.15, *p* < 0.0001 in the sorafenib subgroup and −1.89, IQR −2.46 to −1.21, *p* < 0.0001 in the lenvatinib subgroup), which was also indicated by an increased proportion of patients presenting with ALBI grade 3 at that timepoint (*n* = 28, 36% and *n* = 17, 30% in the sorafenib and lenvatinib subgroup, respectively).

At the end of the entire sequential therapy, the CPS deteriorated by approximately 2 points compared to baseline CPS (2.1 and 1.9 points after sorafenib and lenvatinib therapy, respectively), as shown in Supplementary Materials Figure S4. The median ALBI score at the end of the entire systemic therapy was −1.33 (IQR −1.85 to −0.71) in the sorafenib subgroup, corresponding to ALBI grade 3. In the lenvatinib subgroup, the ALBI score showed no further deterioration compared to the end of first-line therapy (Table 4, Figure S5). Regarding the distribution of ALBI grades at the end of the entire therapy, no significant difference between both subgroups was observed.

### 3.6. Survival

**Figure 2:**
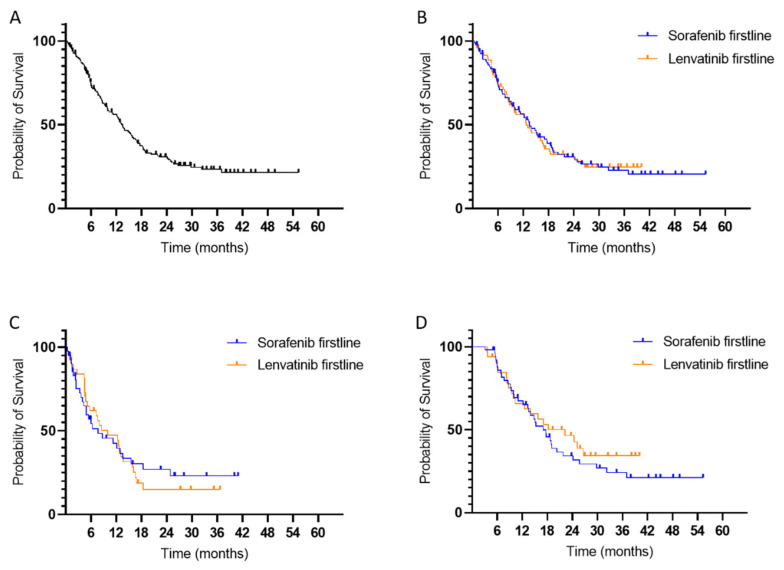
Kaplan-Meier curve of the median overall survival (mOS) from first-line therapy. (**A**) Whole cohort (mOS 410 days); (**B**) sorafenib vs. lenvatinib (mOS sorafenib 411 days; mOS lenvatinib 386 days; *p* value ns); (**C**) patients with only 1 systemic therapy and sorafenib or lenvatinib as first-line therapy (mOS sorafenib 233 days; mOS lenvatinib 302 days; *p* value ns); (**D**): patients with >1 systemic therapy and sorafenib or lenvatinib as first-line therapy (mOS sorafenib 536 days; mOS lenvatinib 676 days; *p* value ns).

The mOS of the entire cohort was 410 days (13.5 months) and did not depend on the choice of first-line therapy, as shown in Figure 2. Patients starting with sorafenib showed an mOS of 411 days (13.5 months). For patients who started with lenvatinib, mOS was 386 days (12.7 months, *p* = not significant (ns)), as shown in Figure 2. In a subgroup analysis only including patients with CPS A, mOS did not differ between patients in the sorafenib or lenvatinib subgroup (Figure S2).

**Figure 3:**
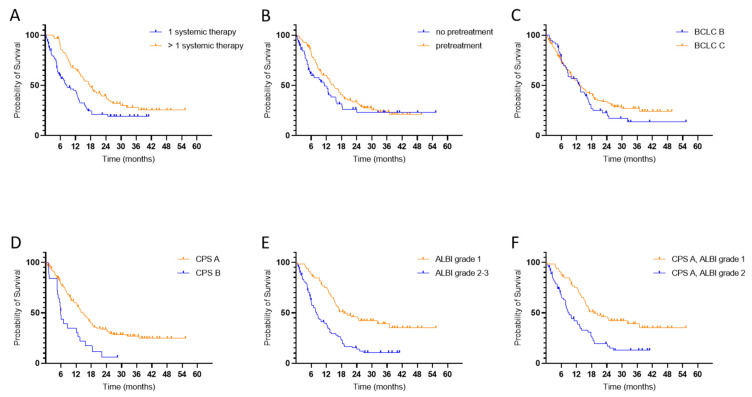
Kaplan-Meier curve of the median overall survival (mOS). (**A**) 1 therapy line vs. >1 therapy line (mOS 1 therapy line 258 days; mOS >1 therapy line 537 days; *p* value 0.0007); (**B**) prior locoregional therapy (mOS no pretreated patients 343 days; mOS pretreated patients 451 days; *p* value ns); (**C**) BCLC B vs. BCLC C (mOS BCLC B 404 days; mOS BCLC C 398 days; *p* value ns); (**D**) patients with CPS A vs. patients with CPS B at baseline (mOS CPS A 451 days; mOS CPS B 182 days; *p* value 0.0005); (**E**) patients with ALBI grade 1 vs. patients with ALBI grade 2 at baseline (mOS ALBI grade 1 567 days; mOS ALBI grade 2–3 243 days; *p* value < 0.0001). (**F**) Patients with CPS A and ALBI grade 1 or ALBI grade 2 at baseline (mOS CPS A, ALBI grade 1 567 days; mOS CPS A, ALBI grade 2 265 days; *p* value < 0.0001).

Patients who received more than 1 systemic therapy had a significantly longer mOS than those who discontinued therapy after the first-line therapy (1 systemic therapy 258 days = 8.5 months vs. sequential treatment 537 days = 17.7 months, *p* = 0.0007), as shown in Figure 3. Patients treated with a sequential systemic treatment presented with a significantly better liver function at baseline. Only 7 patients (9%) receiving more than 1 treatment line suffered from liver cirrhosis with impaired liver function (CPS B) compared to 19 patients (24%) in the group receiving only 1 treatment line. Additionally, patients receiving more than 1 systemic therapy had significantly lower baseline MELD scores (7 points vs. 9 points, *p* = 0.04) and exhibited macrovascular invasion less often (23% vs. 38%, *p* = 0.04), as shown in Table S1.

In most cases, tumor progression was the reason for switching to second-line therapy (Table 2). However, as mentioned above, the choice of first-line therapy, either sorafenib or lenvatinib, did not affect mOS, neither in the group with one systemic therapy nor in the group with sequential treatment (Figure 2). These findings could also be observed in our cohort’s most common first- and second-line sequences (sorafenib-regorafenib, sorafenib-cabozantinib, sorafenib-pembrolizumab and lenvatinib-sorafenib), which resulted in similar mOS (Figure S1).

Patients with impaired liver function at baseline had a significantly poorer mOS (Figure 3). This was also evident in a sub-stratification of patients with CPS A according to ALBI grade (mOS 567 days = 18.6 months vs. 301 days = 9.9 months for CPS A and ALBI grade 1 vs. 2, *p* < 0.0001). Baseline BCLC stage and locoregional pretreatment and surgical tumor resection did not impact mOS both in the whole cohort and the subgroup presenting with preserved liver function at baseline (CPS A), as shown in Figure 3 and Figure S2.

Of note, regarding patients with preserved liver function at baseline, mOS was significantly poorer in patients with impaired ECOG-PS (ECOG PS 0 554 days = 18.2 months vs. ECOG PS 1 298 days = 9.8 months, *p* = 0.0003), as shown in Figure S2. In multivariate analysis, both ALBI grade and ECOG-PS could be identified as independent predictors for mOS. However, CPS could not be identified as an independent prognostic marker (Table S2).

Another important finding is that a longer time on systemic therapy was significantly associated with increased mOS (whole cohort r = 0.8; *p* < 0.0001), as shown in Figure S3.

## 4. Discussion

During the last years, the approval of various regimens for systemic therapy has significantly improved treatment options for patients with advanced HCC. Although, treatment with the combination of atezolizumab and bevacizumab is considered as the new standard of care for first-line treatment of advanced HCC. However, there are still questions about the optimal use and sequence of the various TKIs. In the present real-world study, we therefore evaluated sequences of systemic therapy in patients with advanced HCC for whom first-line therapy with either sorafenib or lenvatinib was initiated.

Our study has revealed several important findings. First, we were able to observe a significant liver function deterioration, represented by the Child-Pugh score and the ALBI score, already during first-line therapy. This was independent of whether the patients received sorafenib or levatinib as first-line therapy. Of note, liver function deterioration was the main reason for discontinuation of systemic therapy, especially in patients who started with sorafenib. Overall, the rate of patients who discontinued systemic therapy was similar during treatment with lenvatinib compared to sorafenib. In our study, only approximately half of the patients received a second-line therapy, without any significant difference between patients who started with sorafenib or lenvatinib. Hatanaka et al. reported similar findings regarding the number of patients eligible for second-line therapy [15,16]. In a study assessing different TKI sequences after discontinuation of an ICI-based first-line therapy, Yoo et al. showed a comparable allocation of patients to subsequent therapeutics to what we observed in our real-world cohort. However, in that study, more patients were eligible for a subsequent second TKI therapy (third line after atezolizumab-bevacicumab). This might be due to a better baseline liver function in comparison to our currently reported cohort [17].

Concerning the total duration of systemic therapy, we observed similar results in patients who received first-line therapy with lenvatinib compared to those who received first-line therapy with sorafenib. In contrast to our experience, treatment duration in the REFLECT trial was longer for first-line therapy with lenvatinib than for first-line therapy with sorafenib [6]. These differences might again be explained by the less favorable baseline characteristics in our real-world cohort, including a relevant proportion of patients with impaired liver function. Furthermore, sorafenib dosage in the real-world setting is frequently reduced to improve tolerability according to the discretion of the treating physician. This underlines the general notion that good therapy tolerance facilitates longer duration of therapy. Hence, it can be assumed that the benefit of the higher antiproliferative activity of lenvatinib over sorafenib may be counterbalanced by better tolerability and longer application of sorafenib in real-world populations. We observed a clear correlation between the duration of TKI treatment and mOS. Similar findings were reported by Hiraoka et. al., with the total duration of therapy and the duration of the different lines of therapy, however, being longer compared to our results. In their study, however, the authors reported the data of a cohort with substantially better baseline liver function as compared to our cohort, which in fact could explain the above-mentioned differences [18].

Regarding the relationship of mOS and choice of first-line therapy, mOS was similar in both patient groups (sorafenib vs. lenvatinib first line). This corresponds to the results of the lenvatinib approval study, where mOS was similar in patients who received either first-line treatment with lenvatinib or with sorafenib, whereas progression-free survival and objective response rates were higher in lenvatinib- vs. sorafenib-treated patients [6].

Patients who were able to undergo sequential therapy showed a significantly longer overall survival compared to those who received only one TKI, accordingly sorafenib or lenvatinib. Of note, patients who received more than one systemic therapy had significantly better liver function at baseline. Previous studies already highlighted this important interrelationship. For example, in a recently published review article, Kudo pointed out that patients with a Child-Pugh score A5, serum albumin level ≥ 3.5 g/dL, and ALBI score ≤ −2.53 or ≤−2.33 have a higher chance of sustaining long-term sequential therapy [19]. Additionally, a severe impact of even slightly impaired liver function has been shown, for example, in a recent post hoc analysis of the SORAMIC-trial, in which patients with a Child-Pugh A5 liver function experienced improved survival after combination therapy with sorafenib and selective internal radiation therapy compared to sorafenib monotherapy while no survival benefit was observed in patients with Child-Pugh A6 [20]. The ALBI grade allows a more accurate subdivision of the Child-Pugh score A5 and thus facilitates improved assessment of liver function. In our cohort, patients with a Child-Pugh score A and ALBI grade 1 presented a significantly longer mOS than patients with a Child-Pugh score A and ALBI grade 2. In a post hoc analysis of the REFLECT trial, patients with ALBI grade 1 at baseline presented a significantly longer mOS compared to those with ALBI grade 2 [21]. The respective mOS was comparable to our study. The relationship between liver function, sequential therapy, and mOS was further highlighted by a retrospective study recently published by Tovoli et al. The investigators were able to demonstrate an mOS of almost 3 years for patients treated with a TKI-based sequential therapy (sorafenib-regorafenib-cabozantinib). This noteworthy survival benefit was, however, limited to patients presenting a well-preserved liver function at the start of third-line therapy [22]. In contrast, if liver function is already impaired, severe adverse events, which can be associated with treatment discontinuation, occur more frequently and patients benefit less from systemic therapy with regard to overall survival [13,23,24].

As mentioned before, liver function deteriorates during TKI treatment, especially in patients with already impaired liver function at the beginning of TKI therapy [15,16]. In our study, liver function, represented by the ALBI score and Child-Pugh score, deteriorated to a similar extent in both patient cohorts who started with sorafenib or lenvatinib. A retrospective analysis by Hatanaka et al. reported similar findings, with a significant deterioration of the ALBI score during treatment with lenvatinib. This was particularly due to a decrease in serum albumin [15]. Hiraoka et al. also reported a significant deterioration of the ALBI score during each therapy line and identified the ALBI score as an independent predictor of mOS. Thus, the ALBI score should be regarded as a cornerstone tool to assess liver function before the initiation of systemic TKI-based therapy [18]. This is further underscored by our current coherent findings, where only ALBI grade but not CPS was identified to be an independent predictor of mOS.

Our study has several limitations. Most importantly, the retrospective design of our study did not allow a head-to-head comparison of the effectiveness of the different treatment algorithms. In this regard, it is a principal difference between sorafenib- and lenvatinib-based first-line therapy that in patients who tolerated sorafenib but progressed during sorafenib, second-line therapy with regorafinib was generally considered standard, whereas the choice of second-line treatment after lenvatinib failure was more flexible. Additionally, our study is based on data derived from a time before ICI-based therapy for HCC was approved. Nevertheless, our real-world data are informative and may help clinicians to guide individual treatment sequences.

## 5. Conclusions

In conclusion, liver function significantly impacts the outcome of systemic therapy. Since systemic therapy can lead to a significant worsening of liver function, it is essential to identify the most suitable therapy sequence for the individual patient with regard to the side effect profile, preservation of liver function, and survival benefit. In this regard, strategies to maintain liver function are important.

## Figures and Tables

**Table 1:** Baseline characteristics of all included patients.

	All Patients (*n* = 164)	Sorafenib as 1st Line (*n* = 93)	Lenvatinib as 1st Line (*n* = 71)	*p* Value (SOR vs. LEN)
Age (years), median (IQR)	68 (60.25–73)	68 (62–72)	66 (57–74)	0.1
Male gender, *n* (%)	140 (85)	81 (87)	59 (83)	0.4
**ECOG PS ^a^**				**0.04**
0, *n* (%)	92 (57)	44 (48)	48 (69)	
1, *n* (%)	64 (40)	44 (48)	20 (29)	
2, *n* (%)	4 (2)	3 (3)	1 (1)	
3, *n* (%)	1 (1)	0 (0)	1 (1)	
Liver cirrhosis, *n* (%)	123 (75)	78 (84)	45 (63)	**0.003**
**Underlying liver disease ^b^**				**0.0003**
ASH, *n* (%)	45 (29)	36 (40)	9 (13)	
NASH, *n* (%)	32 (20)	17 (19)	15 (22)	
HCV, *n* (%)	22 (14)	14 (16)	8 (12)	
HBV, *n* (%)	24 (15)	10 (11)	14 (21)	
Autoimmune, *n* (%)	3 (2)	2 (2)	1 (1)	
Other or cryptogenic, *n* (%)	32 (20)	11 (12)	21 (31)	
**Child-Pugh score ^b^**				0.8
CPS A, *n* (%)	132 (83.5)	72 (83)	60 (84.5)	
CPS B, *n* (%)	26 (16.5)	15 (17)	11 (15.5)	
CPS C, *n* (%)	0	0	0	
**ALBI grade ^c^**				0.1
1, *n* (%)	65 (42)	33 (39)	32 (46)	
2, *n* (%)	85 (55)	47 (55)	38 (54)	
3, *n* (%)	5 (3)	5 (6)	0 (0)	
ALBI score, median (IQR) ^c^	−2.47 (−2.91 to −2.06)	−2.40 (−2.87 to −1.93)	−2.54 (−3.00 to −2.14)	0.46
Albumin (g/dl), median (IQR) ^c^	3.9 (3.5–4.2)	3.8 (3.4–4.2)	4.0 (3.6–4.3)	0.08
Bilirubin (mg/dl), median (IQR) ^b^	1.0 (0.6–1.6)	1.0 (0.6–1.7)	0.9 (0.6–1.5)	0.5
**Ascites, *n* (%) ^d^**	40 (25)	22 (24)	18 (25)	0.8
Refractory ascites, *n* (%)	7 (4)	3 (3)	4 (6)	1.0
Esophageal varices, *n* (%) ^a^	59 (37)	35 (39)	24 (34)	0.4
**Tumor stage ^e^**				0.4
BCLC A, *n* (%)	1 (1)	0 (0)	1 (1)	
BCLC B, *n* (%)	57 (35)	35 (38)	22 (32)	
BCLC C, *n* (%)	104 (63)	57 (61)	47 (67)	
BCLC D, *n* (%)	1 (1)	1 (1)	0	
Macrovascular invasion, *n* (%)	49 (30)	23 (25)	26 (37)	0.1
Extrahepatic tumor manifestations, *n* (%)	80 (49)	43 (46)	37 (52)	0.5
**Prior locoregional therapy, *n* (%)**	106 (65)	60 (65)	46 (65)	0.9
Resection, *n* (%)	29 (18)	16 (17)	13 (18)	0.9
TACE, *n* (%)	55 (34)	30 (32)	25 (35)	0.7
TARE, *n* (%)	55 (34)	32 (34)	23 (32)	0.8

IQR: interquartile range, ECOG PS: Eastern Cooperative Oncology Group performance status, ASH: alcoholic steatohepatitis, NASH: nonalcoholic steatohepatitis, HCV: hepatitis C virus, HBV: hepatitis B virus, CPS: Child-Pugh score, ALBI grade: albumin-bilirubin grade, ALBI score: albumin-bilirubin score, BCLC: Barcelona Clinic Liver Cancer, TACE: transcatheter arterial chemoembolization, TARE: transarterial radioembolization. ^a^ 161 patients included in analysis, ^b^ 158 patients included in analysis, ^c^ 155 patients included in analysis, ^d^ 162 patients included in analysis, ^e^ 163 patients included in analysis.

**Table 2:** Reasons for discontinuation of first-line therapy, sorafenib-based first-line therapy vs. lenvatinib-based first-line therapy.

	Sorafenib (*n* = 88)	Lenvatinib (*n* = 66)
**1 therapy line**	***n* = 40**	***n* = 32**
Progress, *n* (%)	9 (22.5)	3 (9)
Intolerance/Toxicity, *n* (%)	18 (45)	16 (50)
Of that, liver function deterioration, *n* (%)	13 (72)	8 (50)
Of that, other adverse events, *n* (%)	5 (28)	8 (50)
Progress + intolerance, *n* (%)	5 (12.5)	3 (9)
Death, *n* (%)	6 (15)	9 (28)
Other, *n* (%)	2 (5)	1 (3)
**>1 therapy line**	***n* = 48**	***n* = 34**
Progress, *n* (%)	25 (52)	20 (59)
Intolerance/Toxicity, *n* (%)	17 (35)	9 (26)
Of that, liver function deterioration, *n* (%)	6 (35)	3 (33)
Of that, other adverse events, *n* (%)	11 (65)	6 (67)
Progress + intolerance, *n* (%)	5 (10)	4 (12)
Other, *n* (%)	1 (2)	1 (3)

**Table 3:** Duration of sequential systemic therapies according to first-line therapy.

	Sorafenib as 1st-Line (*n* = 93)	Lenvatinib as 1st-Line (*n* = 71)	*p* Value
First-line, days, median (IQR)	99.5 (47–199.5), *n* = 88	120 (44–202.5), *n* = 65	0.3
Second-line, days, median (IQR)	112 (57–175), *n* = 43	92 (37–201.5), *n* = 29	0.3
Third-line, days, median (IQR)	87 (26–143.8), *n* = 12	109 (27.25–192.8), *n* = 12	0.4
Entire therapy, whole cohort, median (IQR)	175.5 (80.5–333), *n* = 74	155 (61.5–395.5), *n* = 57	0.8
Entire therapy, patients with 2 or more systemic therapies, median (IQR)	262 (182–434), *n* = 39	303 (195.5–581.5), *n* = 26	0.7

**Table 4:** Liver function before and after first-line therapy with sorafenib or lenvatinib and at the end of the entire treatment sequence (deviations from 100% are due to rounding).

	Liver Function at Beginning of First-Line Therapy with Sorafenib		Liver Function at the End of First-Line Therapy with Sorafenib		Liver Function at the End of the Entire Sequential Therapy If the Sequence Started with Sorafenib	
**Child**-**Pugh Score**						
	*n* **= 87**		*n* = 77		*n* = 62	
A5, *n* (%)	47 (54)	72 (83)	30 (39)	37 (48)	12 (19)	16 (26)
A6, *n* (%)	25 (29)		7 (9)		4 (7)	
B7, *n* (%)	9 (10)	15 (17)	13 (17)	32 (42)	9 (15)	32 (52)
B8, *n* (%)	4 (5)		9 (12)		8 (13)	
B9, *n* (%)	2 (2)		10 (13)		15 (24)	
C, *n* (%)	0	0	8 (10)	8 (10)	14 (23)	14 (23)
**ALBI Grade/ALBI Score**						
	*n* = 87		*n* = 77		*n* = 62	
1, *n* (%)	35 (40)		14 (18)		4 (6.5)	
2, *n* (%)	47 (54)		35 (46)		23 (37)	
3, *n* (%)	5 (6)		28 (36)		35 (56.5)	
ALBI score, median (IQR)	−2.40 (−2.87 to −1.93)		−1.67 (−2.48 to −1.15)		−1.33 (−1.85 to −0.71)	
	**Liver Function at the Beginning of First-Line Therapy with Lenvatinib**		**Liver Function at the End of First-Line Therapy with Lenvatinib**		**Liver Function at the End of the Entire Sequential Therapy if the Sequence Started with Lenvatinib**	
**Child**-**Pugh score**						
	*n* = 71		*n* = 56		*n* = 35	
A5, *n* (%)	45 (63)	60 (85)	23 (41)	34 (61)	5 (14)	11 (31)
A6, *n* (%)	15 (21)		11 (20)		6 (17)	
B7, *n* (%)	6 (9)	11 (15)	6 (11)	16 (29)	7 (20)	19 (54)
B8, *n* (%)	4 (6)		6 (11)		8 (23)	
B9, *n* (%)	1 (1)		4 (7)		4 (11)	
C, *n* (%)	0	0	6 (11)	6 (11)	5 (14)	5 (14)
**ALBI grade/ALBI score**						
	*n* = 71		*n* = 56		*n* = 38	
1, *n* (%)	33 (46.5)		12 (21)		4 (10.5)	
2, *n* (%)	38 (56.3)		27 (48)		19 (50)	
3, *n* (%)	0		17 (30)		15 (39.5)	
ALBI score, median (IQR)	−2.54 (−3.00 to −2.14)		−1.89 (−2.46 to −1.21)		−1.74 (−2–23 to −0.88)	

## Data Availability

Data analyzed in this study are available from the corresponding author on reasonable request.

## Supplementary Materials

The following supporting information can be downloaded at: https://www.mdpi.com/article/10.3390/cancers14081975/s1, Table S1: Baseline characteristics of patients with only one vs. two or more systemic therapies, Table S2: Univariate and multivariate analysis using Cox regression;Figure S1: Kaplan–Meier curve of the median overall survival (mOS) for patients with more than 1 therapy line with regard to the sequence of first- and second-line therapy; Figure S2: Kaplan Meier Curves of median overall survival (mOS) in a subgroup-analysis including only patients presenting with preserved liver function (CPS A); Figure S3: Correlation analysis between duration of entire TKI treatment and mOS; Figure S4: Liver function deterioration represented by differences in Child-Pugh score; Figure S5: Liver function deterioration as represented by ALBI score.
