# Supplementary material for: Sorafenib Versus Lenvatinib-Based Sequential Systemic Therapy for Advanced Hepatocellular Carcinoma: A Real-World Analysis
Source: Cancers (Basel). 2022 Apr 13;14(8):1975. doi: 10.3390/cancers14081975 (PMC9025688; doi:10.3390/cancers14081975)
Supplement: Supplementary file 1 [file cancers-14-01975-s001.zip › cancers-1669924-supplementary.pdf]

Supplementary Material: Sorafenib Versus Lenvatinib-Based Sequential Systemic Therapy for Advanced Hepatocellular Carcinoma: A Real-World Analysis

Catherine Leyh, Ursula Ehmer, Daniel Rössler, Alexander B. Philipp, Florian P. Reiter, Petia Jeliazkova, Leonie Jochheim, Matthias Jeschke, Janina Hammig, Johannes M. Ludwig, Jens M. Theysohn, Andreas Geier and Christian M. Lange*

Supplemental Materials

Table S1. Baseline characteristics of patients with only one vs. two or more systemic therapies.

	**1 Systemic Therapy (*n* = 80)**	**>1 Systemic Therapy (*n* = 84)**	***p* Value**
Age (years), median (IQR)	66 (59–73)	68.52 (61.25–73)	0.3
Male gender, *n* (%)	66 (82.5)	74 (88)	0.3
**ECOG PS**			0.052
0, *n* (%)	37 (47)	55 (67)	
1, *n* (%)	38 (48)	26 (32)	
2, *n* (%)	3 (4)	1 (1)	
3, *n* (%)	1 (1)	0 (0)	
Liver cirrhosis, *n* (%)	62 (78)	61 (73)	0.5
**Child-Pugh score**			**0.01**
CPS A, *n* (%)	60 (76)	72 (91)	
CPS B, *n* (%)	19 (24)	7 (9)	
CPS C, *n* (%)	0	0	
**ALBI grade**			0.051
1, *n* (%)	27 (34)	38 (50)	
2, *n* (%)	49 (62)	36 (47)	
3, *n* (%)	3 (4)	2 (3)	
Bilirubin (mg/dl), median (IQR)	1.2 (0.7–1.9)	0.8 (0.6–1.4)	0.2
Albumin (g/dl), median (IQR)	3.8 (3.4–4.2)	4.0 (3.6–4.3)	0.053
MELD Score, median (IQR)	9 (7–12)	7 (6–10)	**0.04**
**Ascites, *n* (%)**	25 (31)	15 (18)	0.062
Refractory ascites, *n* (%)	5 (6)	2 (2)	0.1
Esophageal varices, *n* (%)	29 (37)	30 (37)	0.9
**Tumor stage**			0.6
BCLC A, *n* (%)	0 (0)	1 (1)	
BCLC B, *n* (%)	28 (35)	29 (35)	
BCLC C, *n* (%)	52 (65)	53 (64)	
Macrovascular invasion, N (%)	30 (38)	19 (23)	**0.04**
Prior locoregional therapy, N (%)	44 (55)	62 (74)	**0.01**

IQR: interquartile range, ECOG PS: Eastern Cooperative Oncology Group performance status, CPS: Child-Pugh score, ALBI grade: albumin-bilirubin grade, MELD: model of end stage liver disease, BCLC: Barcelona Clinic Liver Cancer.

Table S2. Univariate and multivariate analysis using Cox regression.

Parameter	Univariate Analysis		Multivariate Analysis	
	p Value	Hazard Ratio (95% CI)	*p* value	Hazard Ratio (95% CI)
1st-line Sorafenib vs. Lenvatinib	0.99	1.0 (0.689–1.452)	0.398	1.194 (0.791–1.802)
ECOG PS 1 vs. 0	0.0002	2.075 (1.415–3.042)	0.001	2.018 (1.333–3.055)
Sequential therapy no vs. yes	0.001	1.879 (1.282–2.754)	0.032	1.557 (1.039–2.335)
ALBI grade 2 vs. 1	0.0001	2.489 (1.669–3.710)	0.0001	2.337 (1.511–3.613)
BCLC B vs BCLC C	0.293	1.226 (0.838–1.794)		
CPS B vs A	0.001	2.287 (1.421–3.679)	0.466	1.239 (0.696–2.204
Pretreatment no vs. yes	0.149	0.749 (0.507–1.109)		
Ascites	0.014	1.674 (1-108–2.528)		
Macrovascular invasion	0.006	1.739 (1.171–2.583)		

95% CI: 95% confidence interval, ECOG PS: Eastern Cooperative Oncology Group performance status, CPS: Child-Pugh score, ALBI grade: albumin-bilirubin grade, BCLC: Barcelona Clinic Liver Cancer.

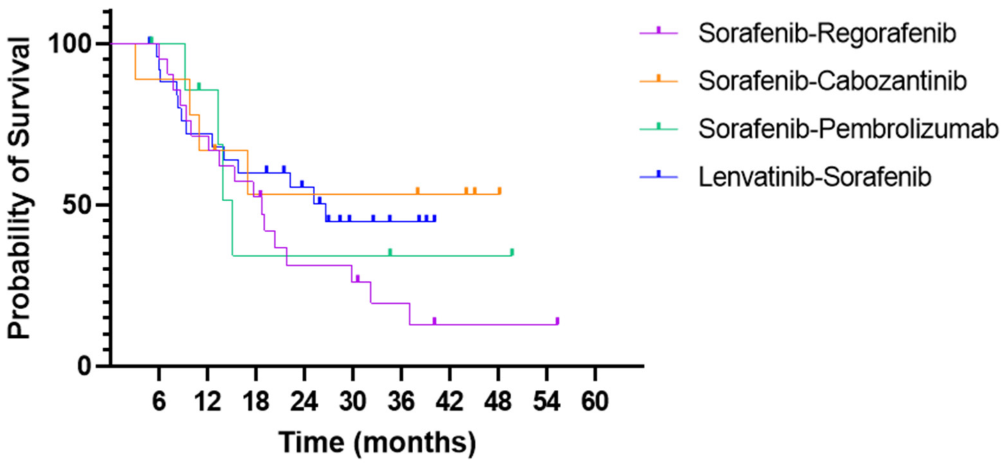

Figure S1. Kaplan Meier Curve of median overall survival (mOS) for patients with more than 1 therapy line with regard to the sequence of 1st and 2nd line therapy. The four most common sequences were analyzed. (mOS Sorafenib-Regorafenib 567 days; mOS Sorafenib-Cabozantinib undefined; mOS Sorafenib-Pembrolizumab 458 days; mOS Lenvatinib-Sorafenib 811 days; *p* value ns)

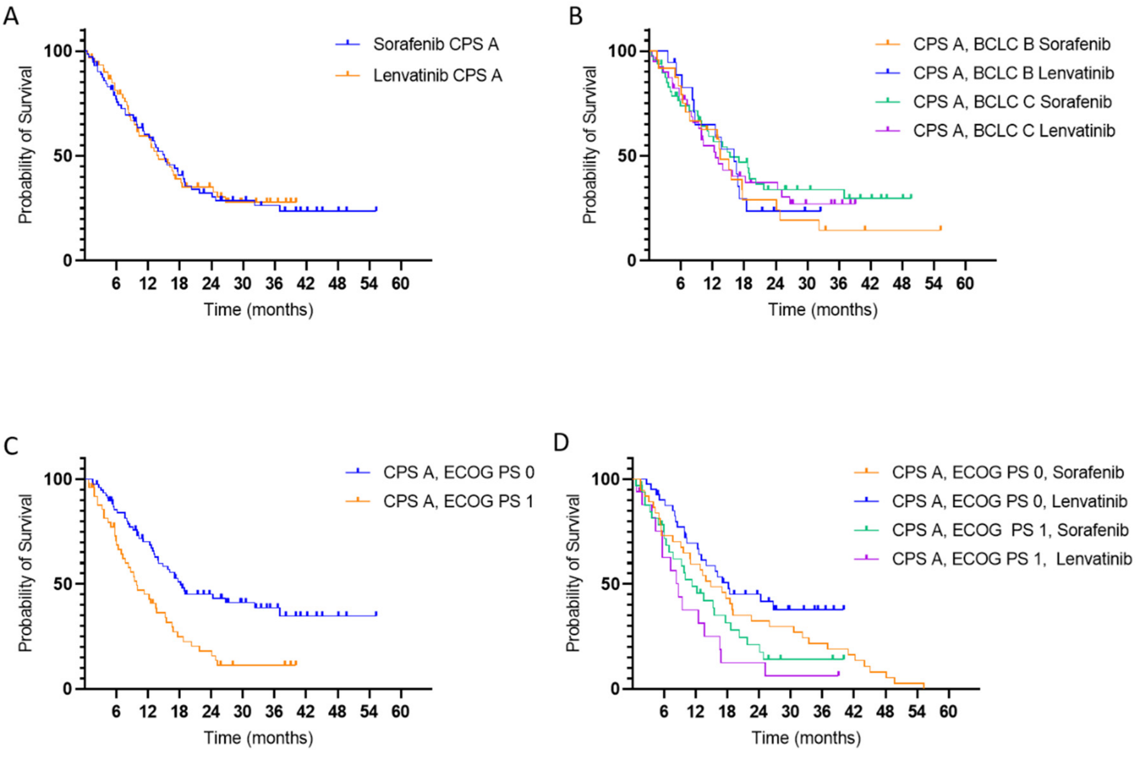

Figure S2. Kaplan-Meier Curves of median overall survival (mOS) in a subgroup-analysis including only patients presenting with preserved liver function (CPS A). (**A**) mOS was not significantly different regarding choice of 1st-line therapy. (**B**) mOS was not significantly different regarding choice of 1st-line and tumor stage. (**C**) Patients with CPS A and better performance status as indicated by ECOG-PS 0 showed significantly better mOS (mOS ECOG PS 0 554 days vs. mOS ECOG PS 1 298 days; *p* value 0.0003). (**D**) Patients with ECOG PS 1 show worse survival as compared to patients with ECOG PS 0 irrespective of choice of 1st-line therapy.

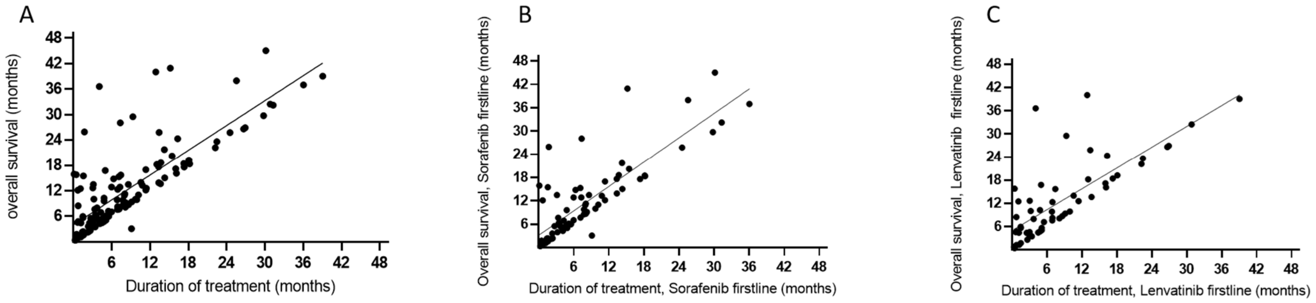

Figure S3. Correlation analysis between duration of entire TKI treatment and mOS. (**A**) whole cohort (r = 0.79, 95% CI 0.7231–0.8512, P value <0.0001); (**B**) Patients starting with Sorafenib as 1st-line (r = 0.76, 95% CI 0.6503–0.8496, *p* value <0.0001). (**C**) Patients starting with Lenvatinib as 1st-line (r = 0.78, 95% CI 0.6499–0.8678, *p* value <0.0001)

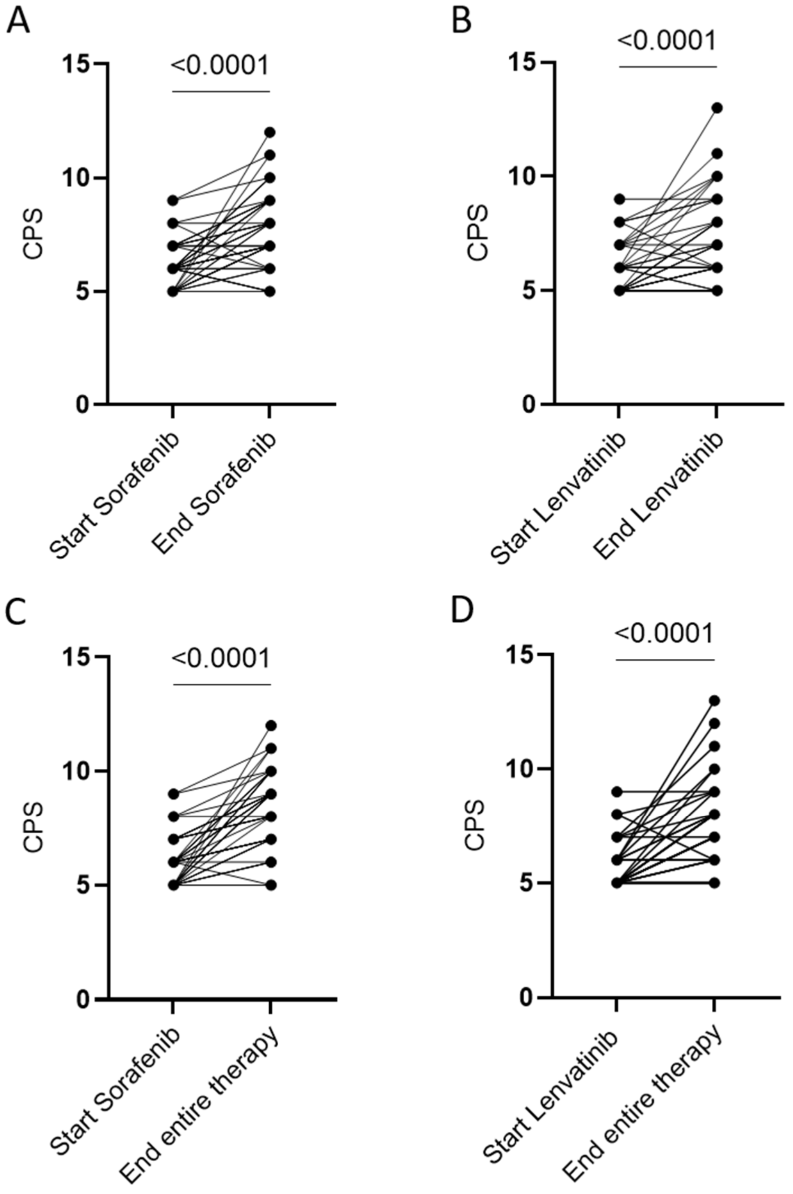

Figure S4 Liver function deterioration represented by differences in Child-Pugh score (**A**) after 1st line therapy with Sorafenib (mean of differences end-start 1.1 points; p value < 0.0001; median treatment duration 99.5 days); (**B**): after 1st line therapy with Lenvatinib (mean of differences end-start 0.9 points; p value < 0.0001; median treatment duration 120 days); (**C**): after the entire systemic therapy when started with Sorafenib (mean of differences end-start 2.1 points; p value <0.0001; median treatment duration 157.5 days); (**D**): after the entire systemic therapy when started with Lenvatinib (mean of differences end-start 1.9 points; p value < 0.0001; median treatment duration 155 days); Y-axis = Child-Pugh score (points)

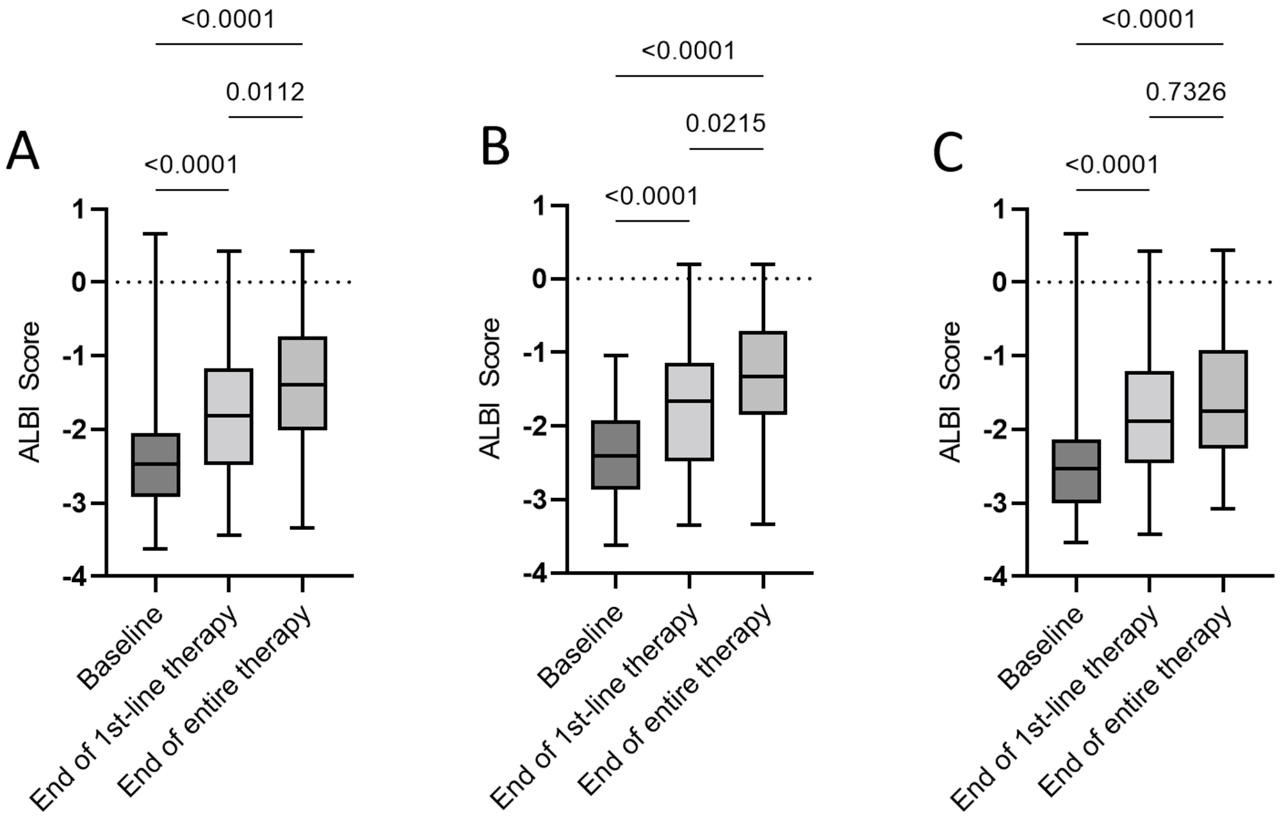

Figure S5. Liver function deterioration as represented by ALBI score. (**A**) Entire cohort. (**B**) Patients with Sorafenib as 1st-line. (**C**). Patients with Lenvatinib as 1st-line.
